# Effect of add-on direct renin inhibitor aliskiren in patients with non-diabetes related chronic kidney disease

**DOI:** 10.1186/1471-2369-13-89

**Published:** 2012-08-23

**Authors:** Szu-yuan Li, Yung-Tai Chen, Wu-Chang Yang, Der-Cherng Tarng, Chih-Ching Lin, Chih-Yu Yang, Wen-Sheng Liu

**Affiliations:** 1 Division of Nephrology, Department of Medicine, Taipei Veterans General Hospital, Taipei, Taiwan; 2 Institute of Clinical of Medicine, National Yang-Ming University, Taipei, Taiwan; 3 Department of Medicine, Taipei City Hospital Heping Fuyou Branch, Taipei, Taiwan; 4 School of Medicine, National Yang-Ming University, Taipei, Taiwan; 5 Department and Institute of Physiology, National Yang-Ming University, Taipei, Taiwan; 6 Division of Nephrology, Department of Medicine, Taipei City Hospital, Zhong-Xing Branch, Taipei, Taiwan

**Keywords:** Aliskiren, Direct renin inhibitor, Proteinuria, CKD

## Abstract

**Background:**

The renin-angiotensin-aldosterone system (RAAS) plays an important role in the progression of chronic kidney disease (CKD). Although dual RAAS inhibition results in worse renal outcomes than monotherapy in high risk type 2 diabetes patients, the effect of dual RAAS inhibition in patients with non-DM CKD is unclear. The aim of this study was to evaluate the potential renoprotective effect of add-on direct renin inhibitor in non-DM CKD patients.

**Methods:**

We retrospectively enrolled 189 non-DM CKD patients who had been taking angiotensin II receptor blockers (ARBs) for more than six months. Patients were divided into an add-on aliskiren group and an ARB monotherapy group. The primary outcomes were a decline in glomerular filtration rate (GFR) and a reduction in urinary protein-to-creatinine ratio at six months.

**Results:**

The baseline characteristics of the two groups were similar. Aliskiren 150 mg daily reduced the urinary protein-to-creatinine ratio by 26% (95% confidence interval, 15 to 37%; p < 0.001). The decline in GFR was smaller in the add-on aliskiren group (−2.1 vs. -4.0 ml/min, p = 0.038). Add-on aliskiren had a neutral effect on serum potassium in the non-DM CKD patients. In subgroup analysis, the proteinuria-reducing effect of aliskiren was more prominent in patients with a GFR less than 60 ml/min, and in patients with a urinary protein-to-creatinine ratio greater than 1.8. The effect of aliskiren in retarding the decline in GFR was more prominent in patients with hypertensive nephropathy than in those with glomerulonephritis.

**Conclusion:**

Add-on direct renin inhibitor aliskiren (150 mg daily) safely reduced proteinuria and attenuated the decline in GFR in the non-DM CKD patients who were receiving ARBs.

## Background

The pathogenesis of chronic kidney disease is multifactorial, and the renin-angiotensin aldosterone system (RAAS) is known to play an important role. The RAAS is the best known regulator of blood pressure (BP), fluid and electrolyte balance through coordinated effects on the heart, blood vessels, and kidneys [1]. When over-activated, the RAAS activates a number of pathways and contributes to the onset and progression of chronic renal damage [2]. Proteinuria is one of the most common findings in chronic kidney disease (CKD) [3]. The amount of urine protein is positively associated with glomerulopathy and tubulointerstitium damage, and is the strongest prognostic factor for renal outcomes in CKD patients [4,5]. Inhibition of the RAAS by angiotensin-converting enzyme (ACE) inhibitors or angiotensin II receptor blockers (ARBs) prevents the progression of CKD in both diabetic and non-DM patients [6-12]. However, in patients with vascular disease or high risk diabetes, dual RAAS inhibition through a combination of ACE inhibitors and ARBs is associated with more adverse events without an increase in benefits compared to monotherapy, as shown by the ONTARGET trial [12]. The ALTITUDE clinical trial attempted to evaluate the potential renoprotective effect of the add-on direct renin inhibitor (DRI) aliskiren in high risk type 2 DM patients who were already taking ACE inhibitors or ARBs, [13] however the trial was terminated early because of adverse events. These two clinical trials clearly indicated that dual RAAS inhibition is not suitable for high risk type 2 DM patients. In contrast, the effect of dual RAAS inhibition in non-DM patients was still unclear. Although the use of add-on DRIs has been shown to be renoprotective in various animal models of non-DM kidney disease, [12,14-17] the clinical implications are still unclear. The aim of current study, therefore, was to evaluate the potential renoprotective effect of the add-on direct renin inhibitor aliskiren in non-DM CKD patients who were already receiving ARB treatment.

## Methods

### Study population

This retrospective study was approved by the Institutional Review Board of Taipei Veterans General Hospital. We enrolled patients who were included in the chronic kidney disease care program at Taipei Veterans General Hospital between March 1, 2010 and April 30, 2011. The CKD care program was launched by the Taiwan Health Promotion Bureau [18]. This program organizes nephrologists, renal nurses and dieticians into a multidisciplinary team to care for CKD patients with the goals of education and clinical protocols. Every patient undergoes regular follow-up visits with clinical evaluation, laboratory examinations, nursing and dietary education every 1–3 months. The CKD care program improves the quality of pre-ESRD care and reduces medical costs [19].

### Inclusion and exclusion criteria

Patients in the CKD care program who were hypertensive, older than 18 years, had non-DM CKD and proteinuria (defined by a spot urinary protein-to-creatinine ratio of >300 mg per gram), and had been taking ARBs for more than 90 days were enrolled. The exclusion criteria were known diabetes mellitus, a urinary protein-to-creatinine ratio of more than 3500 mg per gram, an estimated glomerular filtration rate (GFR) of less than 15 ml per minute per 1.73 m2 of body-surface area, [20] chronic urinary-tract infections, a serum potassium level greater than 6.0 mmol per liter at the time of enrollment, and kidney transplant recipients. Patients who received add-on aliskiren 150 mg daily were defined as the aliskiren group, and the remainders were defined as the ARB monotherapy group. In the aliskiren group, patients who had not been taking aliskiren for more than 90 days were also excluded.

### Measurement of urine protein and renal function

The patients were examined initially and at 8, 16, and 24 weeks. Blood pressure, concomitant medications, the results of laboratory tests, and urinary protein-to-creatinine ratios were assessed at each visit. The urinary protein concentration was determined by immunoturbidimetry, and the creatinine concentration was determined by means of the Jaffe reaction. The Modification of Diet in Renal Disease (MDRD) formula was used to estimate the glomerular filtration rate [20]. All of the other laboratory variables were also measured centrally in a CAP qualified laboratory. The primary outcome was the percentage reduction in the urinary protein-to-creatinine ratio and the secondary outcome was the eGFR decline from baseline to the end of follow-up.

### Statistical analysis

Normally distributed continuous data are expressed as means ± standard deviations. Numeric data with non-normal distributions are expressed as medians and interquartile ranges. To compare the parameters among each group, Pearson *χ*^2^ tests were carried out for categorical variables, and the independent *t*-test and Mann–Whitney *U* test were used for parametric and nonparametric continuous variables, respectively. To compare the parameters within groups, the paired *T*-test and Wilcoxon test were used for parametric and nonparametric continuous variables, respectively. All probabilities were two tailed. A *p*-value less than 0.05 was considered significant.

## Results

Add on aliskiren attenuated urinary protein and decline in estimated glomerular filtration rate in non-DM CKD patients

**Figure 1:**
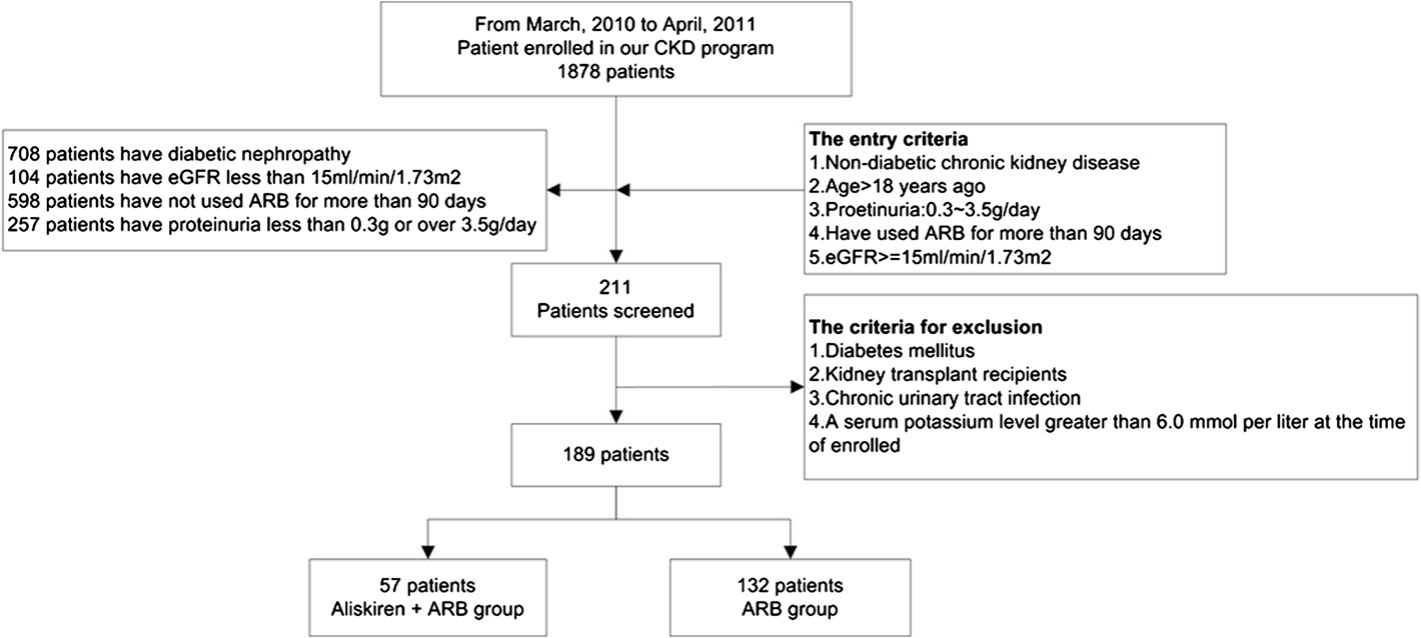
Patient selection flowchart.

Fifty-seven patients in the aliskiren group and 132 patients in the ARB monotherapy group were enrolled in this study (Figure 1). Among the enrolled patients, 78.9% took Losartan 160 mg and 21.1% took irbesartan 300 mg daily, there are no difference between aliskiren and ARB alone group in ARB type and dosage. The baseline demographic data including age, sex, mean urinary protein-to-creatinine ratio, estimated glomerular filtration rate (eGFR), and blood pressure of the two groups were comparable (Table 1) .

**Table 1:** Baseline patient characteristics

**Characteristic**	**Aliskiren + ARB Group**	**ARB Group**	**P value**
	**N = 57**	**N = 132**	
Demographic			
Age	63.9 ± 19.3	66.5 ± 15.4	0.367
Male gender	61.4% (n = 35)	68.9% (n = 91)	0.313
Clinical			
Body-mass index	24.8 ± 3.9	25.2 ± 3.7	0.548
Mean sitting blood pressure-mmHg			
Systolic	134.9 ± 16.6	131.0 ± 14.0	0.123
Diastolic	77.2 ± 10.8	77.4 ± 10.5	0.135
Urine protein-to-creatinine ratio	1.84 (0.89 ∼ 2.77)	1.81 (0.84 ∼ 2.78)	0.723
Estimated glomerular filtration rate ml/min/1.73 m2	50.9 ± 31.1	42.5 ± 19.9	0.062
Hemoglobin (g/liter)	12.0 ± 1.9	11.8 ± 1.9	0.525
Chronic kidney disease stage			0.173
Stage I	10.5% (n = 6)	4.5% (n = 6)	
Stage II	17.5% (n = 10)	7.6% (n = 10)	
Stage IIIA	14.0% (n = 8)	23.5% (n = 31)	
Stage IIIB	28.1% (n = 16)	36.4% (n = 48)	
Stage IV	29.8% (n = 17)	28.0% (n = 37)	
Etiology			0.917
Hypertensive nephropathy	36.8% (n = 21)	34.1% (n = 45)	
Chronic glomerulonephritis	43.9% (n = 25)	47.0% (n = 62)	
Interstitial nephritis	19.3% (n = 11)	18.9% (n = 25)	
Triglycerides (mg/dl)	146.3 ± 100.2	133.0 ± 79.4	0.369
Cholesterol (mg/dl)			
Total	198.9 ± 52.4	183.7 ± 45.7	0.062
Low-density lipoprotein	116.4 ± 38.8	112.4 ± 37.7	0.567
High-density lipoprotein	51.8 ± 14.0	48.6 ± 17.3	0.309
Serum potassium (mmol/liter)	4.2 ± 0.5	4.4 ± 0.6	0.037
Antihypertensive drugs received at baseline			
Calcium-channel blocker	52.6% (n = 30)	43.9% (n = 58)	0.272
Beta-blocker	21.1% (n = 12)	15.9% (n = 21)	0.393
Diuretic	35.1% (n = 20)	47.0% (n = 62)	0.130
Alpha-blocker	14.0% (n = 8)	5.3% (n = 7)	0.042

**Figure 2:**
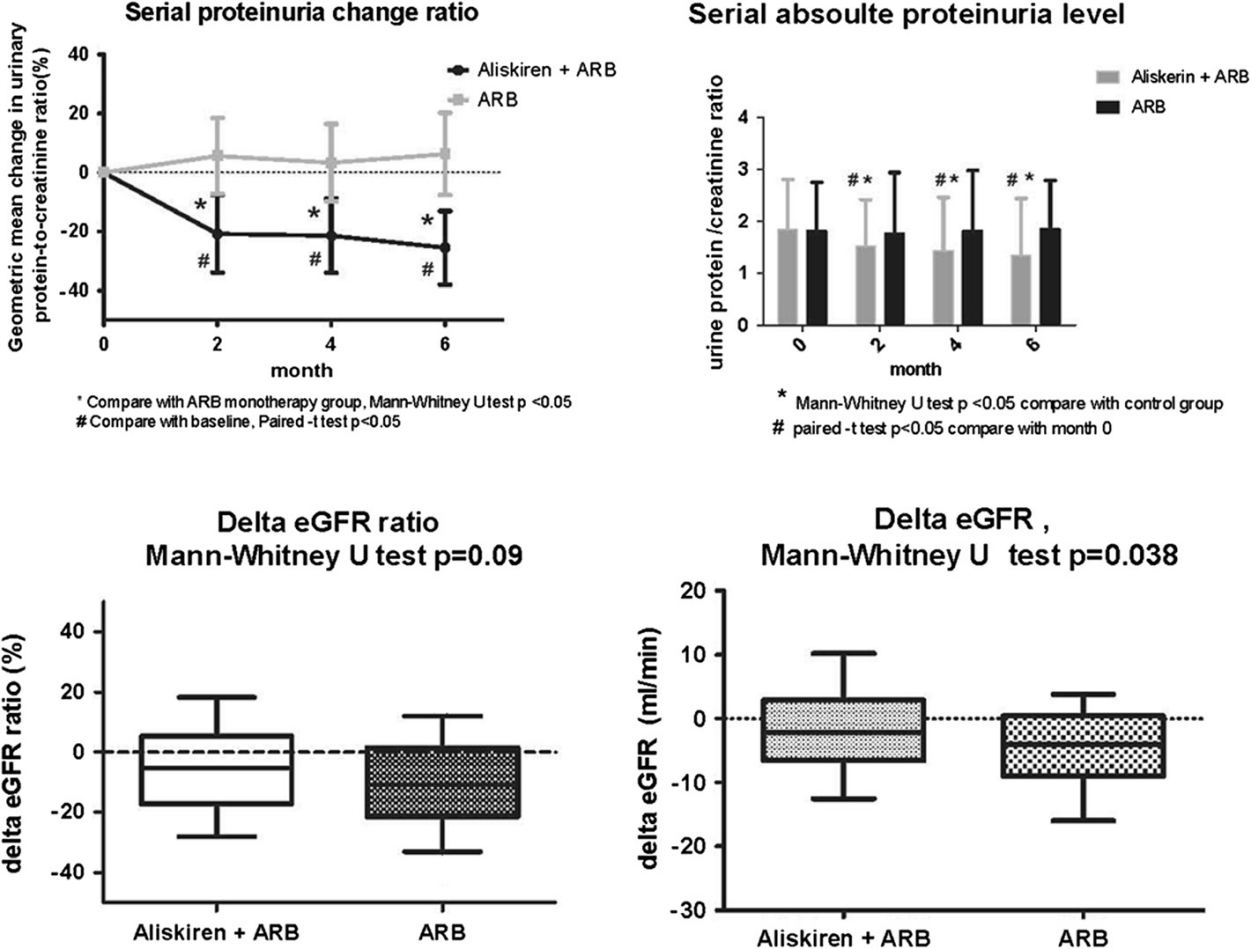
**Serial change of proteinuria and eGFR in the two groups.** In the 6-month study period, add-on aliskiren reduced the urinary protein-to-creatinine ratio and slowed down the decline in GFR decline. * Compared with the ARB group, Mann–Whitney *U* test p < 0.05; # Compared with baseline, paired *t* test p < 0.05.

By the end of the study, compared with baseline, there was no significant change of mean blood pressure between the two groups (−1.9 mmHg in the aliskiren group and −1.3 mmHg in the ARB monotherapy group, p = 0.802). After the 6 months follow-up period, add-on aliskiren treatment had reduced the mean urinary protein-to-creatinine ratio by 26% (95% confidence interval [CI], 15-37%; p < 0.001), while there was no significant change in the amount of urine protein in the ARB monotherapy group. The decline in eGFR was slower in the aliskiren group compared to the ARB monotherapy group (−2.1 vs. -4.0 ml/min, p = 0.038) (Figure 2). With regards to the risk of hyperkalemia, add-on aliskiren had a neutral effect on serum potassium. No stroke or myocardial infarction events occurred during the study period in either group.

### Subgroup analysis for anti-proteinuric effect and retarded GFR decline rate

**Figure 3:**
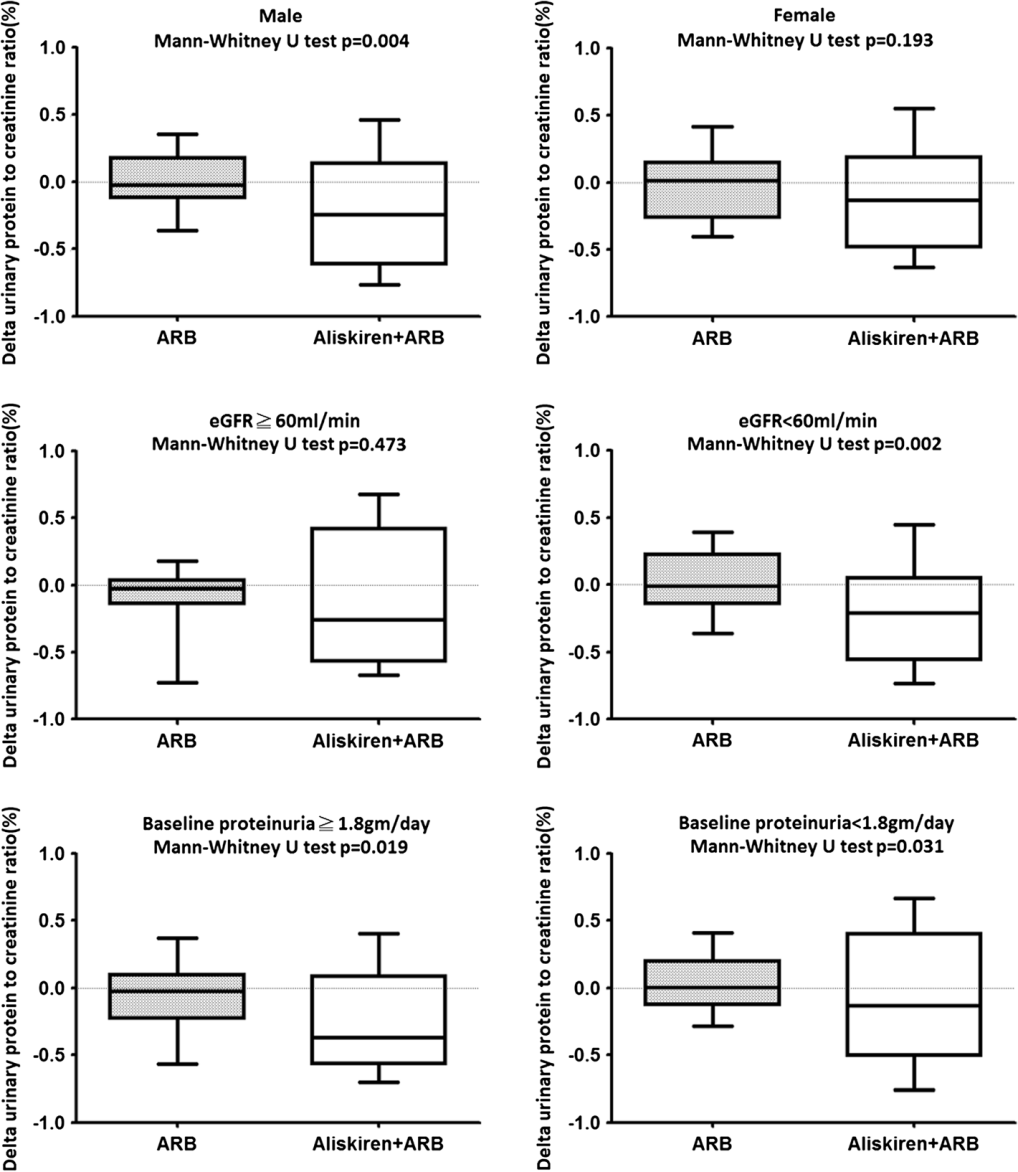
**Subgroup analysis for the anti-proteinuric effect of aliskiren.** The anti-proteinuric effect of add-on aliskiren was analyzed by gender, baseline eGFR and proteinuria.

To evaluate the anti-proteinuric effect of aliskiren in different patient populations, we performed subgroup analysis stratified by gender, baseline eGFR and urine protein. The proteinuria-reducing effect of add-on aliskiren was more prominent in males, and in patients with a GFR less than 60 ml/min. Aliskiren had an anti-proteinuric effect in non-DM CKD patients regardless of the urinary protein amount, but the effect was more prominent in patients with heavier proteinuria (Figure 3). In subgroup analysis for a decline in eGFR, add-on aliskiren retarded the decline in GFR in patients with hypertensive nephropathy (p = 0.037) but not in patient with glomerulonephritis ( p = 0.40).

## Discussion

In diabetic and non-DM nephropathy, blocking the RAAS is the mainstay of therapy to prevent progression of renal disease [21,22]. ACE inhibitors and ARB have been shown to have a renoprotective effect and reduced morbidity and mortality in patients with chronic kidney disease [22,23] However, the strategy of dual RAAS inhibition seems to have different clinical impacts in diabetic and non-diabetic CKD patients. In high risk and type 2 diabetic CKD patients, a combination of ACE inhibitors and ARBs is associated with more adverse renal events [12]. Although add-on DRIs have been shown to reduce urinary protein in DM nephropathy patients who are receiving ARBs, [24] a large-scale clinical trial evaluating the effect of add-on DRI aliskiren 300 mg in high risk DM patients who were taking ACI inhibitors or ARBs [13] was terminated early because of increased adverse events, including non-fatal stroke, hypotension, hyperkalemia and renal complications. However, the potential renoprotective effect of dual RAAS inhibition has not been investigated in non-DM CKD patients. In the current study, we found that add-on DRI aliskiren 150 mg daily safely reduced proteinuria and attenuated the decline in GFR in non-DM CKD patients who were already taking ARBs.

The major difference between the ALTITUDE and the current study are the patient population and the dosage of aliskiren. ALTITUDE study included patients taking ACE inhibitors but the AVOID study and the current study did not. Because ACE inhibitor related cough is prevalent in Asians, most patients who need RAAS inhibition receive ARBs rather than ACE inhibitors in our CKD program, thus, we enrolled patients taking ARBs in current study. In Taiwan, the medical expenditure is paid by National Health Insurance Program, which covers 99.9% Taiwanese population. Currently, aliskiren can be claimed 150 mg (1 tablet) daily. In contrast, patients in the ALTITUDE trial took aliskiren 300 mg daily, and have a higher possibility of hyperkalemia and other side effects.

Similar with previous studies on diabetic nephropathy, [25,26] our results showed that the proteinuria reducing effect of add-on aliskiren was more prominent in the high risk group. We also found that the anti-proteinuric effect was more prominent in patients with an eGFR of less than 60 ml/min, and in patients with a urinary protein-to-creatinine ratio greater than 1.8. In addition, we found that add-on aliskiren attenuated the decline in GFR in hypertensive nephropathy patients. Although not reaching statistical significance, our results suggest that add-on aliskiren may have a greater renoprotective effect in early stage CKD patients (GFR > 60 ml/min and not profound proteinuria). There was no significant change in mean blood pressure after add-on aliskiren (p = 0.263), indicating that add-on DRIs have a renoprotective effect in non-DM CKD patients beyond a blood pressure lowering effect. This finding was also confirmed in several non-DM CKD animal models [14,27,28]. In the AVOID trial, the reduction in systolic blood pressure was only 2 mmHg after add-on aliskiren treatment in the study group.In ONGARTET study, combine RAAS blockade also has marginal effect on the reduction of blood pressure. The patients in the current study took half of the dosage of aliskiren than the patients in the AVOID study. This may explain why aliskiren had less of a blood pressure lowering effect in our study. Although renin inhibitor monotherapy provides a dose-dependent BP lowering effect, [29] our study and the AVOID trial showed that add-on aliskiren in CKD patients receiving ARBs had a lesser effect on blood pressure.

The renoprotective effect of add-on aliskiren in patients with non-DM CKD has been evaluated in clinical settings in several studies, however all have had limitations. Without a control group, Lopez et al. and Tang et al. demonstrated that add-on aliskiren reduced urinary protein in kidney transplant recipients (n = 12) and immunoglobulin A nephropathy patients (n = 25), respectively [30][31]. A prospective trial conducted by Tsukasa Nakamura et al. found that combination therapy with aliskiren and ARBs decreased proteinuria in non-DM CKD patients more effectively than monotherapy alone, however their findings did not reach statistical significance due to the limited number of patients (n = 12 in each group) [32]. By studying 189 non diabetic CKD patients, the present study clearly shows that aliskiren reduced proteinuria in patients with non-DM chronic kidney disease who were receiving ARBs. Add-on aliskiren (150 mg daily) for six months reduced the mean urinary protein-to-creatinine ratio by 26%.

There are several limitations to the current study. First, it was not a randomized prospective control trial, and the results may have been biased by patient selection and data retrieval. Second, the 6-month study period was not long enough to reveal uncommon side effects. Third, plasma renin activity was not examined in our study, thus, it is unknown whether changes of plasma renin activity are correlated with the renoprotective effect of aliskiren. Even with these limitations, we believe our results are reliable and suggest important clinical implications. The patients enrolled in this study were selected from a registered CKD program, with frequent follow-up visits and the best clinical care, and their medical data were complete. To the best of our knowledge, the current study is the largest scale study to evaluate the effect of an add-on direct renin inhibitor in non-DM CKD patients.

## Conclusion

In conclusion, we found that add-on aliskiren (150 mg daily) in non-DM CKD patients already receiving ARBs safely reduced proteinuria and attenuated the decline in GFR. The anti-proteinuric effect was more prominent in patients with an estimated glomerular filtration rate less than 60 ml/min, and in patients with a urinary protein-to-creatinine ratio greater than 1.8. Further prospective studies are necessary to confirm these findings.

## Competing interest

The authors declare that they have no competing interests.

## Authors’ contributions

SY Li and YT Chen designed research, performed, and wrote the paper; WC Yang and DC Tarng directed the study; CC Lin, CY Yang, and WS Liu interpreted the data. All authors read and approved the final manuscript.

## Pre-publication history

The pre-publication history for this paper can be accessed here:

http://www.biomedcentral.com/1471-2369/13/89/prepub
